# Supplementary material for: A within-host infection model to explore tolerance and resistance
Source: eLife. 2025 Feb 13;14:e104052. doi: 10.7554/eLife.104052 (PMC12143882; doi:10.7554/eLife.104052)
Supplement: Source code 1. [file elife-104052-code1.zip › Duneau_WHD_2024_Rmarkdown.html]

HR / PLUD analysis


Code 

- Show All Code
- Hide All Code

# HR / PLUD analysis

#### Jean-Baptiste Ferdy

#### 23/06/2023

# 1 Wounds increase both susceptibility to infection and PLUD

Four lines taken from the DGRP (RAL584, RAL559, RAL818 and RAL630)
are injected with *P. rettgeri*. In one treatment they are
“wounded” (ie pricked) before being injected with the bacterial
suspension. In another (our control) they are injected with no prior
wound. The model predicts that the wound should increase both mortality
and the PLUD.

```
rm(list=ls())
d_surv_wound <- read.table(file="wound_SURVIVAL.csv",header=TRUE)
d_surv_wound$Line <- factor(d_surv_wound$Line)
d_surv_wound$Day_of_injection <- factor(d_surv_wound$Day_of_injection)
d_cfu_wound  <- read.table(file="wound_CFU.csv",header=TRUE)
genotype <- c("584","559","818","630")
```

## 1.1 Survival analysis

```
library(survival)
with(subset(d_surv_wound,Dose=="PBS"),tapply(Censor,list(Line,Treatment),mean,na.rm=TRUE))
```

```
##      No_wound     wound
## 559 0.0877193 0.3888889
## 584 0.1971831 0.3625000
## 630 0.2105263 0.5287356
## 818 0.2391304 0.3826087
```

```
with(subset(d_surv_wound,Dose=="P_rettgeri"),tapply(Censor,list(Line,Treatment),mean,na.rm=TRUE))
```

```
##      No_wound     wound
## 559 0.4218750 0.9609610
## 584 0.8701299 0.9269841
## 630 0.7741935 0.9964029
## 818 0.4457831 0.9960630
```

```
require(coxme)
m_full <- coxme(Surv(t,Censor)~Dose*Line*Treatment+(1|Day_of_injection),data=subset(d_surv_wound,Line %in% genotype))
m_1 <- coxme(Surv(t,Censor)~Dose+Line*Treatment+(1|Day_of_injection),data=subset(d_surv_wound,Line %in% genotype))
anova(m_full,m_1)
```

```
## Analysis of Deviance Table
##  Cox model: response is  Surv(t, Censor)
##  Model 1: ~Dose * Line * Treatment + (1 | Day_of_injection)
##  Model 2: ~Dose + Line * Treatment + (1 | Day_of_injection)
##   loglik  Chisq Df P(>|Chi|)    
## 1 -13268                        
## 2 -13290 45.243  7 1.227e-07 ***
## ---
## Signif. codes:  0 '***' 0.001 '**' 0.01 '*' 0.05 '.' 0.1 ' ' 1
```

The wound by itself reduces survival, but it has a stronger impact in
case the fly is infected: wound increases susceptibility to the
infection.

```
library(survival)
msurv <- survfit(Surv(t,Censor)~Line+Treatment,data=subset(d_surv_wound,Dose!="PBS"))
dmsurv <- data.frame(Line=rep(rep(genotype,each=2),msurv$strata),
                     Treatment=rep(rep(c("no_wound","wound"),length(genotype)),msurv$strata),
                     time=msurv$time,proportion=msurv$surv)
dmsurv <- rbind(data.frame(Line=rep(genotype,each=2),
                           Treatment=rep(c("no_wound","wound"),length(genotype)),
                           time=0,proportion=1),dmsurv)
symbol <- 21:24
names(symbol) <- genotype
plot(proportion~time,data=dmsurv,type="n",las=1,xlab="Hours post injection",ylab="Proportion surviving")
foo <- sapply(split(dmsurv,with(dmsurv,paste(Line,Treatment))),function(xx) lines(proportion~time,
                                                  data=xx,type="b",
                                                  cex=0.75,pch=symbol[as.character(Line)],
                                                  lty=ifelse(xx$Line=="PBS",2,1),
                                                  col=ifelse(xx$Treatment=="wound","indianred","steelblue"),
                                                  bg=adjustcolor(ifelse(xx$Treatment=="wound","indianred","steelblue"),alpha.f=0.75)))
points(0,1,pch=21,col="gray",bg="gray",cex=1.25)
nw <- with(subset(d_surv_wound,Dose!="PBS" & Treatment=="wound"),tapply(Censor,Line,length))
nnw <- with(subset(d_surv_wound,Dose!="PBS" & Treatment=="No_wound"),tapply(Censor,Line,length))
l1 <- legend("topright",legend=paste(genotype," (N=",nnw[genotype],"/",nw[genotype],")",sep=""),
             pch=symbol,bty="n")
legend(100,l1$rect$top,legend=c("No wound","Wound"),
       fill=adjustcolor(c("steelblue","indianred"),alpha.f=0.75),bty="n",xjust=1)
```

Cox proportionnal hazard model, with Treatment and Line considered
has a fixed effects. ‘Day\_of\_injection’ corresponds to replicate
experiments and defines here a random block.

```
require(coxme) 
(m_full <- coxme(Surv(t,Censor)~Line*Treatment+(1|Day_of_injection),data=subset(d_surv_wound,Dose=="P_rettgeri" & Line %in% genotype)))
```

```
## Cox mixed-effects model fit by maximum likelihood
##   Data: subset(d_surv_wound, Dose == "P_rettgeri" & Line %in% genotype)
##   events, n = 1730, 2133
##   Iterations= 9 58 
##                     NULL Integrated    Fitted
## Log-likelihood -12138.08  -11348.47 -11338.37
## 
##                     Chisq    df p     AIC     BIC
## Integrated loglik 1579.24  8.00 0 1563.24 1519.59
##  Penalized loglik 1599.43 11.65 0 1576.13 1512.59
## 
## Model:  Surv(t, Censor) ~ Line * Treatment + (1 | Day_of_injection) 
## Fixed coefficients
##                              coef  exp(coef)  se(coef)     z       p
## Line584                 1.0789510  2.9415922 0.1218001  8.86 0.0e+00
## Line630                 0.8043381  2.2352165 0.1253731  6.42 1.4e-10
## Line818                 0.1029432  1.1084284 0.1366904  0.75 4.5e-01
## Treatmentwound          2.3317672 10.2961207 0.1141234 20.43 0.0e+00
## Line584:Treatmentwound -0.5460156  0.5792532 0.1456456 -3.75 1.8e-04
## Line630:Treatmentwound -0.4727406  0.6232918 0.1494860 -3.16 1.6e-03
## Line818:Treatmentwound  0.5792169  1.7846404 0.1622703  3.57 3.6e-04
## 
## Random effects
##  Group            Variable  Std Dev   Variance 
##  Day_of_injection Intercept 0.4282655 0.1834114
```

```
m_1 <- coxme(Surv(t,Censor)~Line+Treatment+(1|Day_of_injection),data=subset(d_surv_wound,Dose=="P_rettgeri" & Line %in% genotype))
m_0 <- coxme(Surv(t,Censor)~Line+(1|Day_of_injection),data=subset(d_surv_wound,Dose=="P_rettgeri" & Line %in% genotype))

#LRT
anova(m_full,m_1)
```

```
## Analysis of Deviance Table
##  Cox model: response is  Surv(t, Censor)
##  Model 1: ~Line * Treatment + (1 | Day_of_injection)
##  Model 2: ~Line + Treatment + (1 | Day_of_injection)
##   loglik  Chisq Df P(>|Chi|)    
## 1 -11348                        
## 2 -11385 72.592  3 1.221e-15 ***
## ---
## Signif. codes:  0 '***' 0.001 '**' 0.01 '*' 0.05 '.' 0.1 ' ' 1
```

```
anova(m_1,m_0)
```

```
## Analysis of Deviance Table
##  Cox model: response is  Surv(t, Censor)
##  Model 1: ~Line + Treatment + (1 | Day_of_injection)
##  Model 2: ~Line + (1 | Day_of_injection)
##   loglik  Chisq Df P(>|Chi|)    
## 1 -11385                        
## 2 -12084 1397.4  1 < 2.2e-16 ***
## ---
## Signif. codes:  0 '***' 0.001 '**' 0.01 '*' 0.05 '.' 0.1 ' ' 1
```

Wounded flies die faster than non wounded ones, but the effect of
wound varies among the different D. melanogaster lines.

Another Cox proportionnal hazard model, with Line this time
considered has a random factor. As above, ‘Day\_of\_injection’ defines
random block.

```
require(coxme)
coxme(Surv(t,Censor)~Treatment+(1|Line)+(1|Day_of_injection),data=subset(d_surv_wound,Dose=="P_rettgeri" & Line %in% genotype))
```

```
## Cox mixed-effects model fit by maximum likelihood
##   Data: subset(d_surv_wound, Dose == "P_rettgeri" & Line %in% genotype)
##   events, n = 1730, 2133
##   Iterations= 16 84 
##                     NULL Integrated    Fitted
## Log-likelihood -12138.08  -11391.52 -11374.78
## 
##                     Chisq   df p     AIC     BIC
## Integrated loglik 1493.12 3.00 0 1487.12 1470.75
##  Penalized loglik 1526.61 8.55 0 1509.51 1462.87
## 
## Model:  Surv(t, Censor) ~ Treatment + (1 | Line) + (1 | Day_of_injection) 
## Fixed coefficients
##                    coef exp(coef)  se(coef)     z p
## Treatmentwound 2.153131  8.611782 0.0608317 35.39 0
## 
## Random effects
##  Group            Variable  Std Dev    Variance  
##  Line             Intercept 0.28757287 0.08269815
##  Day_of_injection Intercept 0.42411099 0.17987013
```

The conclusion is unchanged.

## 1.2 Analysis of PLUD

### 1.2.1 Estimating the PLUD

It is not possible to count more than few tens of CFU in a single
droplet: above that number, colonies overlap and rapidly become
indistinguishable. Our counts are therefore right truncated, with values
above 50 or so considered as infinite. Using a simple Poisson model on
such CFU counts would yield under estimated PLUD values. To solve this
issue, we use instead a binomial/Poisson mixture i.e. a model which
likelihood combines the probability to observe more than 50 CFU in a
droplet and the Poisson probability to observe k CFU when k<50.

```
require(CFUfit)
plud <-  with(d_cfu_wound,
              loadEstimate(BACTERIA,
                      1/(4*Dilution),
                      Volume,
                      sample_ID,
                      maxCFU = 50,
                      ignoreAboveMaxCFU = FALSE,
                      logbase = 10))

pos <- match(plud$sampleID,d_surv_wound$sample_ID)
plud$Day_of_injection <- d_surv_wound[pos,"Day_of_injection"]
plud$Sex       <- factor(d_surv_wound[pos,"Sex"])
plud$Line      <- factor(d_surv_wound[pos,"Line"])
plud$Plate     <- factor(d_surv_wound[pos,"Plate"])
plud$Treatment <- factor(d_surv_wound[pos,"Treatment"])
plud$Dose      <- d_surv_wound[pos,"Dose"]
plud$Replicate <- d_surv_wound[pos,"Replicate"]
plud$t         <- d_surv_wound[pos,"t"]
```

### 1.2.2 Detecting outliers

In this experiment, some flies may have died from the wound, rather
than from the infection. In these flies, the PLUD should be very much
lower than in other flies, and most importantly it would not reflect the
interaction between the fly immunity and the bacterial pathogen. These
flies could thus bias our analysis and should be removed from the
dataset. To solve this potential issue, we use a Rosner test to detect
PLUD outliers, which we can further ingore in our analysis. The Rosner
test is done per combination Line x Treatment, all experiment replicates
pooled.

```
library(EnvStats)
plud$outlier <- FALSE
plud$grpe <- with(plud,factor(paste(Line,Treatment,sep="_")))
for(g in levels(plud$grpe)) {
    l <- plud$grpe == g & !is.na(plud$logload)
    test <- try(rosnerTest(plud$logload[l],k = floor(sum(l,na.rm=TRUE)/2)))
    if("try-error" %in% class(test)) {
        plud$outlier[l] <- NA
    } else {
        plud$outlier[which(l)[test$all.stats$Obs.Num[test$all.stats$Outlier]]] <- TRUE
    }
}
```

The list of potential outliers

```
with(plud,table(grpe,outlier))
```

```
##               outlier
## grpe           FALSE TRUE
##   559_No_wound    29    7
##   559_wound       48    4
##   584_No_wound    74    0
##   584_wound       65    2
##   630_No_wound    44    1
##   630_wound       47    0
##   818_No_wound    40    0
##   818_wound       28    0
```

### 1.2.3 Is the PLUD increased when the fly is wounded prior to infection?

Distribution of PLUD for each Line/Treatment. Crosses indicate the
potential outliers detected by the Rosner test: they all correspond to
low PLUD values.

```
require(beanplot)
require(beeswarm)

gr <- beanplot(logload~Treatment+factor(Line,levels=genotype),
               ylab="",ylim=c(2,8),
               col=list(adjustcolor("steelblue",alpha.f=0.75),adjustcolor("indianred",alpha.f=0.75)),
               what=c(F,T,T,F),
               axes=FALSE,
               data=subset(plud, Line %in% genotype & !outlier))
axis(1,2*(1:4)-0.5,gsub("wound.","",gr$names[c(F,T)]))
axis(2,with(plud,pretty(logload)),las=1)
title(ylab="log_10 PLUD",xlab="D. melanogaster DGRP line")
box()
pos <- 1:8; names(pos) <- gr$names
with(subset(plud, Line %in% genotype),
     beeswarm(logload~pos[paste(Treatment,Line,sep=".")],
              pwpch = ifelse(outlier,4,21),
              cex=0.5,
              bg="white",add=T))
#n <- with(subset(dPLUDest,!is.na(PLUDest) & Sex=="Male" & Line %in% genotype),tapply(PLUDest,list(Treatment,Line),length))
n <- with(subset(plud,!is.na(logload) & Sex=="Male" & Line %in% genotype & logload>0),tapply(logload,factor(Line,levels=genotype),length))
mtext(text=paste0("(N=",as.numeric(n),")"),side=3,line=0,at=2*(1:4)-0.5)
abline(v=(0:4)*2+0.5,lty=2)
```

Is the PLUD increased when the fly is wounded prior to infection, as
the model predicts? We adjust a Gaussian model to logload estimates,
with a random block effect added as in the survival analysis.
Observations are weighed according to the inverse squared standard error
of PLUD estimates, so that imprecise PLUD estimates are less influential
in model fitting. Goodness of fit is assessed using DHARMa.

```
require(spaMM)
require(DHARMa)

## first analysis: outliers are not removed
m_full <- fitme(logload~Treatment*Line+(1|Day_of_injection),
                prior.weights=1/(se.logload^2),
                data=subset(plud,Line %in% genotype))

## goodness of fit
sim <- simulateResiduals(fittedModel = m_full,refit = TRUE) 
plot(sim) ## no surprise: problems with outliers...
```

Test of the interaction Treatment x Line

```
m_noInt <- fitme(logload~Treatment+Line+(1|Day_of_injection),
                prior.weights=1/(se.logload^2),
                data=subset(plud,Line %in% genotype))
anova(m_full,m_noInt)
```

```
##      chi2_LR df   p_value
## p_v 1.902321  3 0.5929256
```

Test of the Treatment effect

```
m_noTr <- fitme(logload~Line+(1|Day_of_injection),
                prior.weights=1/(se.logload^2),
                data=subset(plud,Line %in% genotype))
anova(m_noTr,m_noInt)
```

```
##      chi2_LR df      p_value
## p_v 11.04414  1 0.0008896792
```

**Conclusion:** on average, the wound does increase the
PLUD, with no significant difference among lines.

Same analysis than above, but with outliers removed: potential
outliers with extremely low PLUD being detected in the control only,
removing them will necessarily reduce the significance of the wound
effect.

```
## second analysis: outliers are removed
m_full <- fitme(logload~Treatment*Line+(1|Day_of_injection),
                prior.weights=1/(se.logload^2),
                data=subset(plud,Line %in% genotype & !outlier))

## Goodness of fit
## sim <- simulateResiduals(fittedModel = m_full,refit = TRUE) 
## plot(sim) ## all is good!

m_noInt <- fitme(logload~Treatment+Line+(1|Day_of_injection),
                prior.weights=1/(se.logload^2),
                data=subset(plud,Line %in% genotype & !outlier))

m_noTr <- fitme(logload~Line+(1|Day_of_injection),
                prior.weights=1/(se.logload^2),
                data=subset(plud,Line %in% genotype & !outlier))

#test of interaction
anova(m_full,m_noInt)
```

```
##      chi2_LR df  p_value
## p_v 1.257257  3 0.739307
```

```
#test of treatment
anova(m_noTr,m_noInt)
```

```
##      chi2_LR df      p_value
## p_v 11.29718  1 0.0007762495
```

**Conclusion:** the global effect remains significant
when all potential outliers are removed.

## 1.3 Computing CI for log HR and log PR

Bootstrap CI are computed both for log HR and log PLUD ratio. The
models used here are writen so that the wound effect is estimated for
each line, with not further computation required (while it would be
obtained by adding the wound effect to the interaction term in the
models used above).

```
library(boot)
dtmp <- subset(d_surv_wound,Line %in% genotype)
dtmp <- dtmp[c("t","Censor","Line","Treatment","Day_of_injection")]
dtmp <- na.omit(dtmp)
m <- coxme(Surv(t,Censor)~-1+Line+Line:Treatment+(1|Day_of_injection),data=dtmp)
cox.fun <- function(d) {mtmp <- update(m,data=d); return(mtmp$coef)}
bcox <- censboot(dtmp, cox.fun, R = 499,strata=dtmp$Line) ## sample size per Line must stay constant

ci <- list()
for(l in as.character(c(559,584,630,818))) 
  ci[[l]] <- boot::boot.ci(bcox,conf=0.95,type="perc",
                           index=grep(paste0(l,":Treatmentwound"),names(bcox$t0)))

d.lHR <- data.frame(lHR=sapply(ci,function(xx) xx$t0),
                    lHR.inf=sapply(ci,function(xx) xx$percent[4]),
                    lHR.sup=sapply(ci,function(xx) xx$percent[5]))
row.names(d.lHR) <- names(ci)

m_full <- fitme(logload~-1+Line+Line:Treatment+(1|Day_of_injection),
                prior.weights=1/(se.logload^2),
                data=subset(plud,Line %in% genotype & !outlier))
ci <- list()
for(l in as.character(c(559,584,630,818))) ci[[l]] <- confint(m_full,parm=paste0("Line",l,":Treatmentwound"),
                                                              boot_args=list(nsim=499, seed=123))
```

```
## Bootstrap replicates:  bootstrap took 20.91 s.
## BOOTSTRAP CONFIDENCE INTERVAL CALCULATIONS
## Based on 499 bootstrap replicates
## 
## CALL : 
## boot.ci(boot.out = list(499, "parametric"), conf = 0.95, type = c("basic", 
## "perc", "norm"), t0 = 0.122716219227553, `str(long numeric 't')` = " num [1:499] 0.0587 0.1227 0.1269 0.2423 0.19 ...")
## 
## Intervals : 
## Level      Normal              Basic              Percentile     
## 95%   (-0.0263,  0.2692 )   (-0.0189,  0.2699 )   (-0.0245,  0.2643 )  
## Calculations and Intervals on Original Scale
## Bootstrap replicates:  bootstrap took 20.45 s.
## BOOTSTRAP CONFIDENCE INTERVAL CALCULATIONS
## Based on 499 bootstrap replicates
## 
## CALL : 
## boot.ci(boot.out = list(499, "parametric"), conf = 0.95, type = c("basic", 
## "perc", "norm"), t0 = 0.0942071969585529, `str(long numeric 't')` = " num [1:499] 0.1222 -0.035 0.0294 0.0788 0.1096 ...")
## 
## Intervals : 
## Level      Normal              Basic              Percentile     
## 95%   (-0.0173,  0.2036 )   (-0.0156,  0.2033 )   (-0.0149,  0.2041 )  
## Calculations and Intervals on Original Scale
## Bootstrap replicates:  bootstrap took 21.07 s.
## BOOTSTRAP CONFIDENCE INTERVAL CALCULATIONS
## Based on 499 bootstrap replicates
## 
## CALL : 
## boot.ci(boot.out = list(499, "parametric"), conf = 0.95, type = c("basic", 
## "perc", "norm"), t0 = 0.190544439936448, `str(long numeric 't')` = " num [1:499] 0.2793 0.1687 0.2697 0.0647 0.2855 ...")
## 
## Intervals : 
## Level      Normal              Basic              Percentile     
## 95%   ( 0.0369,  0.3284 )   ( 0.0307,  0.3336 )   ( 0.0475,  0.3504 )  
## Calculations and Intervals on Original Scale
## Bootstrap replicates:  bootstrap took 19.9 s.
## BOOTSTRAP CONFIDENCE INTERVAL CALCULATIONS
## Based on 499 bootstrap replicates
## 
## CALL : 
## boot.ci(boot.out = list(499, "parametric"), conf = 0.95, type = c("basic", 
## "perc", "norm"), t0 = 0.0908069965505851, `str(long numeric 't')` = " num [1:499] -0.0202 -0.1107 0.1036 -0.0713 0.3123 ...")
## 
## Intervals : 
## Level      Normal              Basic              Percentile     
## 95%   (-0.0836,  0.2915 )   (-0.0985,  0.2976 )   (-0.1160,  0.2801 )  
## Calculations and Intervals on Original Scale
```

```
d.lPR <- data.frame(lPR=sapply(ci,function(cc) cc$t0),
                    lPR.inf=sapply(ci,function(cc) cc$percent[4]),
                    lPR.sup=sapply(ci,function(cc) cc$percent[5]))
rownames(d.lPR) <- names(ci)

d.lHR.lPR <- cbind(d.lHR,d.lPR)
print(d.lHR.lPR)
```

```
##          lHR   lHR.inf  lHR.sup       lPR     lPR.inf   lPR.sup
## 559 1.971306 1.7799341 2.214779 0.1227162 -0.02445795 0.2643366
## 584 1.094615 0.9378026 1.258762 0.0942072 -0.01485025 0.2040637
## 630 1.358437 1.2090762 1.533215 0.1905444  0.04745684 0.3504179
## 818 1.669848 1.4742311 1.875761 0.0908070 -0.11598426 0.2801091
```

### 1.3.1 log PLUD ratio as a function of log HR

Plotting the estimated log PLUD ratio as a function of estimated log
HR.

```
plot(lPR~lHR,
     data=d.lHR.lPR,
     xlim=c(0,2),ylim=c(-0.1,0.3),type="p",las=1,
     xlab="log HR relative to no wound",
     ylab="log PLUD ratio relative to no wound")
abline(h=0,lty=2)
abline(v=0,lty=2)
with(d.lHR.lPR,segments(lHR.inf,lPR,lHR.sup,lPR,col="indianred"))
with(d.lHR.lPR,segments(lHR,lPR.inf,lHR,lPR.sup,col="indianred"))
points(lPR~lHR,data=d.lHR.lPR,pch=21,col="white",bg="white",cex=2)
points(lPR~lHR,data=d.lHR.lPR,pch=21,
       col="indianred",bg=adjustcolor("indianred",alpha.f=0.75))
points(0,0,pch=21,col="white",bg="white",cex=2)
points(0,0,pch=21,bg=adjustcolor("steelblue",alpha.f=0.75),col="steelblue")
with(d.lHR.lPR,text(lHR,lPR,rownames(d.lHR.lPR),adj=c(1.5,-1),offset = 1))
text(0,0,"no wound",adj=c(-0.2,-1))
```

Mortality is significantly increased in all lines, while the PLUD
significantly increases only in line 630. Keep in mind that the previous
analysis found no significant interaction between Line and Treatment
effetcs, meaning that Line do not differ in how they react to the wound.
The lack of significance in three out of four lines therefore probably
reflects a lack of power when lines are analyzed separatly.

# 2 Deletion of immune effectors increase susceptibility to infection and PLUD

We now analyse mortality and PLUD in lines that are deleted for some
effectors of the immune defenses. Namely the deleted effectors are:

- **BOM**: bomanins
- **A**: defensins
- **B**: drosocin, attacins, diptericins
- **AB**: defensins, drosocin, attacins, diptericins (ie
  effectors of both A and B)

As bomanins are ineffective against *P. rettgeri*, we will use
the BOM line as a control. The model predicts that the PLUD should be
higher in A, B and AB than in BOM. The analysis follows the same global
logic as in the previous section.

```
rm(list=ls())
d_surv_abab <- read.table(file="ABAB_SURVIVAL.csv",header=TRUE)
d_cfu_abab  <- read.table(file="ABAB_CFU.csv",header=TRUE)
genotype <- c("BOM","A","B","AB")
color <- c(BOM="steelblue1",A="steelblue",B="indianred2",AB="indianred3")
d_surv_abab$Line <- factor(d_surv_abab$Line,levels=genotype)
d_cfu_abab$Line <- factor(d_cfu_abab$Line,levels=genotype)
```

## 2.1 Survival analysis

```
library(survival)
msurv <- survfit(Surv(time,Dead)~factor(Line,genotype),data=subset(d_surv_abab,Line %in% genotype & Sex=="M"))
dsurv <- data.frame(Line=rep(genotype,msurv$strata),time=msurv$time,proportion=msurv$surv)
dsurv <- rbind(data.frame(Line=genotype,time=0,proportion=1),dsurv)
plot(proportion~time,data=dsurv,type="n",las=1,xlab="Hours post injection",ylab="Proportion surviving")
foo<-sapply(split(dsurv,dsurv$Line),function(xx) lines(proportion~time,
                                                  data=xx,type="b",
                                                  cex=0.75,
                                                  pch=21,col=color[as.character(xx$Line)],
                                                  bg=adjustcolor(color[as.character(xx$Line)],alpha.f=0.75)))
points(0,1,pch=21,col="gray",bg="gray",cex=1.25)
n <- with(subset(d_surv_abab,Sex=="M"),tapply(Dead,Line,length))
legend("topright",legend=paste(genotype," (N=",n[genotype],")",sep=""),pch=21,col=color,pt.bg=adjustcolor(color,alpha.f=0.75),bty="n")
```

Cox proportionnal hazard model. ‘Box’ defines a random block which
correspond to replicate experiments.

```
require(coxme)
with(d_surv_abab,table(Box,Line))
```

```
##    Line
## Box BOM  A  B AB
##   1  56 30 55 70
##   2  57 49 63 53
```

```
(m_full <- coxme(Surv(time,Dead)~factor(Line)+(1|Line/Box),data=subset(d_surv_abab,Line %in% genotype)))
```

```
## Cox mixed-effects model fit by maximum likelihood
##   Data: subset(d_surv_abab, Line %in% genotype)
##   events, n = 411, 433
##   Iterations= 15 78 
##                     NULL Integrated    Fitted
## Log-likelihood -2147.054  -2077.907 -2070.867
## 
##                    Chisq  df p    AIC    BIC
## Integrated loglik 138.30 5.0 0 128.30 108.20
##  Penalized loglik 152.38 5.9 0 140.57 116.85
## 
## Model:  Surv(time, Dead) ~ factor(Line) + (1 | Line/Box) 
## Fixed coefficients
##                     coef exp(coef)  se(coef)    z       p
## factor(Line)A  0.5974276  1.817438 0.2869476 2.08 3.7e-02
## factor(Line)B  1.6605497  5.262203 0.2854766 5.82 6.0e-09
## factor(Line)AB 1.3672942  3.924717 0.2829205 4.83 1.3e-06
## 
## Random effects
##  Group    Variable    Std Dev      Variance    
##  Line/Box (Intercept) 0.2366127040 0.0559855717
##  Line     (Intercept) 0.0128127447 0.0001641664
```

```
anova(m_full)
```

```
## Analysis of Deviance Table
##  Cox model: response is Surv(time, Dead)
## Terms added sequentially (first to last)
## 
##               loglik Chisq Df Pr(>|Chi|)    
## NULL         -2147.1                        
## factor(Line) -2077.9 138.3  3  < 2.2e-16 ***
## ---
## Signif. codes:  0 '***' 0.001 '**' 0.01 '*' 0.05 '.' 0.1 ' ' 1
```

Removing A and/or B effectors significantly reduces survival compared
to removing BOM.

## 2.2 Analysis of PLUD

Estimation of the PLUD and detection of potential outliers.

```
require(CFUfit)
plud <-  with(subset(d_cfu_abab,Sex=="M"),
              loadEstimate(Bacteria,
                      1/(4*Dilution),
                      Volume,
                      sample_ID,
                      maxCFU = 50,
                      ignoreAboveMaxCFU = FALSE,
                      logbase = 10))

pos <- match(plud$sampleID,d_surv_abab$sample_ID)
plud$Sex       <- d_surv_abab[pos,"Sex"]
plud$Line      <- d_surv_abab[pos,"Line"]
plud$Box     <- d_surv_abab[pos,"Box"]
plud$t         <- d_surv_abab[pos,"t"]

library(EnvStats)
plud$outlier <- FALSE
plud$grpe <- with(plud,factor(paste(Line,Box,sep="_")))
for(g in levels(plud$grpe)) {
    l <- plud$grpe == g & !is.na(plud$logload)
    test <- try(rosnerTest(plud$logload[l],k = floor(sum(l,na.rm=TRUE)/2)))
    if("try-error" %in% class(test)) {
        plud$outlier[l] <- NA
    } else {
        plud$outlier[which(l)[test$all.stats$Obs.Num[test$all.stats$Outlier]]] <- TRUE
    }
}

with(plud,table(grpe,outlier))
```

```
##        outlier
## grpe    FALSE TRUE
##   A_1      25    0
##   A_2      31    0
##   AB_1     45    0
##   AB_2     39    1
##   B_1      41    0
##   B_2      39    1
##   BOM_1    31    1
##   BOM_2    25    0
```

```
require(beanplot)
require(beeswarm)

gr <- beanplot(logload~Line,
               #ylab="log10(PLUD)",
               ylim=c(4.25,7),
               col=as.list(adjustcolor(color,alpha.f=0.75)),
               what=c(F,T,T,F),
               axes=TRUE,
               data=subset(plud,!outlier))
title(ylab="log_10 PLUD",xlab="D. melanogaster DGRP line")
box()
pos <- 1:4; names(pos) <- gr$names
with(plud,
     beeswarm(logload~Line,
              pwpch = ifelse(outlier,4,21),
              cex=0.75,
              bg="white",add=T))
n <- with(subset(plud,!is.na(logload)),tapply(logload,factor(Line,levels=genotype),length))
mtext(text=paste0("(N=",as.numeric(n),")"),side=3,line=0,at=1:4)
```

We now compare the PLUD between lines, using the same type of mixed
effect model as before.

```
require(spaMM)
require(DHARMa)

# In principle the random effect should be Line/Box but this poses convergence problems in spaMM.
# We therefore create a unique ID for each box, combining Line and Box. This should be equivalent 
# and is better handled by spaMM.
plud$BBox <- factor(with(plud,paste(Line,Box,sep="_")))

## first analysis: outliers are not removed
m_full <- fitme(logload~Line+(1|BBox),
                prior.weights=1/(se.logload^2),
                data=plud)
summary(m_full)
```

```
## formula: logload ~ Line + (1 | BBox)
## ML: Estimation of lambda and phi by ML.
##     Estimation of fixed effects by ML.
## family: gaussian( link = identity ) 
##  ------------ Fixed effects (beta) ------------
##             Estimate Cond. SE t-value
## (Intercept)   5.9184  0.04193 141.132
## LineA         0.2154  0.05732   3.759
## LineB         0.2923  0.05345   5.468
## LineAB        0.2375  0.05281   4.497
##  --------------- Random effects ---------------
## Family: gaussian( link = identity ) 
##            --- Variance parameters ('lambda'):
## lambda = var(u) for u ~ Gaussian; 
##    BBox  :  3.894e-07  
##              --- Coefficients for log(lambda):
##  Group        Term Estimate Cond.SE
##   BBox (Intercept)   -14.76   23.81
## # of obs: 279; # of groups: BBox, 8 
##  -------------- Residual variance  ------------
## Prior weights: 535.492 216.34 408.246 518.746 573.743 ...
## Coefficients for log(phi)  ~ 1  :
##             Estimate Cond. SE
## (Intercept)    3.611  0.08467
## Estimate of phi=residual var:  36.99 
##  ------------- Likelihood values  -------------
##                         logLik
## logL       (p_v(h)): -67.91228
```

```
## Goodness of fit
sim <- simulateResiduals(fittedModel = m_full,refit = TRUE) 
plot(sim) ## significant deviation from normality and outliers detected
```

```
## remove outliers and refit
m_full <- fitme(logload~Line+(1|BBox),
                prior.weights=1/(se.logload^2),
                data=subset(plud,!outlier))
summary(m_full)
```

```
## formula: logload ~ Line + (1 | BBox)
## ML: Estimation of lambda and phi by ML.
##     Estimation of fixed effects by ML.
## family: gaussian( link = identity ) 
##  ------------ Fixed effects (beta) ------------
##             Estimate Cond. SE t-value
## (Intercept)   5.9369  0.03921 151.414
## LineA         0.1970  0.05344   3.685
## LineB         0.2893  0.05002   5.784
## LineAB        0.2287  0.04934   4.635
##  --------------- Random effects ---------------
## Family: gaussian( link = identity ) 
##            --- Variance parameters ('lambda'):
## lambda = var(u) for u ~ Gaussian; 
##    BBox  :  1.218e-06  
##              --- Coefficients for log(lambda):
##  Group        Term Estimate Cond.SE
##   BBox (Intercept)   -13.62   16.87
## # of obs: 276; # of groups: BBox, 8 
##  -------------- Residual variance  ------------
## Prior weights: 535.492 216.34 408.246 518.746 573.743 ...
## Coefficients for log(phi)  ~ 1  :
##             Estimate Cond. SE
## (Intercept)    3.464  0.08513
## Estimate of phi=residual var:  31.94 
##  ------------- Likelihood values  -------------
##                         logLik
## logL       (p_v(h)): -46.71321
```

```
## Goodness of fit
sim <- simulateResiduals(fittedModel = m_full,refit = TRUE) 
plot(sim) ## all is good!
```

```
m_noLine <- fitme(logload~1+(1|BBox),
                prior.weights=1/(se.logload^2),
                data=subset(plud,!outlier))

#test of Line differences
anova(m_full,m_noLine)
```

```
##      chi2_LR df      p_value
## p_v 17.01371  3 0.0007021673
```

The differences of PLUD among lines are highly signficant.

## 2.3 PLUD as a function of HR

Computing confidence interval relative to BOM.

```
library(boot)
dtmp <- subset(d_surv_abab,Line %in% genotype)
dtmp <- dtmp[c("t","Dead","Line","Box")]
dtmp <- na.omit(dtmp)
m <- coxme(Surv(t,Dead)~Line+(1|Line/Box),data=dtmp)
cox.fun <- function(d) {mtmp <- update(m,data=d); return(mtmp$coef)}
bcox <- censboot(dtmp, cox.fun, R = 499,strata=dtmp$Line) ## sample size per Line must stay constant

ci <- list()
for(l in c("A","B","AB"))
  ci[[l]] <- boot::boot.ci(bcox,conf=0.95,type="perc",
                           index=match(paste0("Line",l),names(bcox$t0)))

d.lHR <- data.frame(lHR=sapply(ci,function(xx) xx$t0),
                    lHR.inf=sapply(ci,function(xx) xx$percent[4]),
                    lHR.sup=sapply(ci,function(xx) xx$percent[5]))
row.names(d.lHR) <- names(ci)

dtmp <- subset(plud,!is.na(logload))
m_full <- fitme(logload~Line+(1|BBox),
                prior.weights=1/(se.logload^2),
                data=dtmp)
ci <- list()
for(l in c("A","B","AB")) ci[[l]] <- confint(m_full,parm=paste0("Line",l),
                                                  boot_args=list(nsim=499, seed=123))
```

```
## Bootstrap replicates:  bootstrap took 21.38 s.
## BOOTSTRAP CONFIDENCE INTERVAL CALCULATIONS
## Based on 499 bootstrap replicates
## 
## CALL : 
## boot.ci(boot.out = list(499, "parametric"), conf = 0.95, type = c("basic", 
## "perc", "norm"), t0 = 0.2154489540364, `str(long numeric 't')` = " num [1:499] 0.195 0.261 0.208 0.157 0.17 ...")
## 
## Intervals : 
## Level      Normal              Basic              Percentile     
## 95%   ( 0.1116,  0.3278 )   ( 0.1102,  0.3302 )   ( 0.1007,  0.3207 )  
## Calculations and Intervals on Original Scale
## Bootstrap replicates:  bootstrap took 26.25 s.
## BOOTSTRAP CONFIDENCE INTERVAL CALCULATIONS
## Based on 499 bootstrap replicates
## 
## CALL : 
## boot.ci(boot.out = list(499, "parametric"), conf = 0.95, type = c("basic", 
## "perc", "norm"), t0 = 0.292270521454085, `str(long numeric 't')` = " num [1:499] 0.307 0.331 0.369 0.285 0.331 ...")
## 
## Intervals : 
## Level      Normal              Basic              Percentile     
## 95%   ( 0.1873,  0.4002 )   ( 0.1896,  0.4085 )   ( 0.1760,  0.3949 )  
## Calculations and Intervals on Original Scale
## Bootstrap replicates:  bootstrap took 28.49 s.
## BOOTSTRAP CONFIDENCE INTERVAL CALCULATIONS
## Based on 499 bootstrap replicates
## 
## CALL : 
## boot.ci(boot.out = list(499, "parametric"), conf = 0.95, type = c("basic", 
## "perc", "norm"), t0 = 0.237499095762843, `str(long numeric 't')` = " num [1:499] 0.218 0.284 0.267 0.174 0.203 ...")
## 
## Intervals : 
## Level      Normal              Basic              Percentile     
## 95%   ( 0.1359,  0.3431 )   ( 0.1324,  0.3429 )   ( 0.1321,  0.3426 )  
## Calculations and Intervals on Original Scale
```

```
d.lPR <- data.frame(lPR=sapply(ci,function(cc) cc$t0),
                    lPR.inf=sapply(ci,function(cc) cc$percent[4]),
                    lPR.sup=sapply(ci,function(cc) cc$percent[5]))
rownames(d.lPR) <- names(ci)

d.lHR.lPR <- cbind(d.lHR,d.lPR)
d.lHR.lPR
```

```
##          lHR   lHR.inf   lHR.sup       lPR   lPR.inf   lPR.sup
## A  0.5974276 0.2967716 0.9728855 0.2154490 0.1006821 0.3207335
## B  1.6605497 1.3721468 2.0443531 0.2922705 0.1760055 0.3949256
## AB 1.3672942 1.1041061 1.7484437 0.2374991 0.1320862 0.3426159
```

Estimated log PLUD ratio as a function of estimated log HR.

```
plot(lPR~lHR,
     data=d.lHR.lPR,
     xlim=c(0,2),ylim=c(-0.1,0.4),type="p",las=1,
     xlab="log HR relative to BOM",
     ylab="log PLUD ratio relative to BOM")
abline(h=0,lty=2)
abline(v=0,lty=2)
with(d.lHR.lPR,segments(lHR.inf,lPR,lHR.sup,lPR,col=color[-1]))
with(d.lHR.lPR,segments(lHR,lPR.inf,lHR,lPR.sup,col=color[-1]))
points(lPR~lHR,data=d.lHR.lPR,pch=21,col="white",bg="white",cex=2)
points(lPR~lHR,data=d.lHR.lPR,pch=21,
       col=color[-1],bg=adjustcolor(color[-1],alpha.f=0.75))
points(0,0,pch=21,col="white",bg="white",cex=2)
points(0,0,pch=21,bg=adjustcolor("steelblue",alpha.f=0.75),col="steelblue")
with(d.lHR.lPR,text(lHR,lPR,rownames(d.lHR.lPR),adj=c(-0.5,-1),offset = 1))
text(0,0,"BOM",adj=c(-0.2,-1))
```

# 3 Deleting Diptericin only is sufficient to increase both susceptibility and the PLUD

In the previous approach, we have used mutants that have several
immune effectors suppressed. To confirm the fact that it is really the
decrease in defense capacity and hence of resistence, which is
responsible from the increase in PLUD, we have performed the same sort
of experiments in mutants with only one type of effector deleted. More
precisely, we have compares the effect of deleting

- **Diptericins**  the most active effector against
  *P. rettgeri*
- **Drosomycin** an AMP which as no effect against *P.
  rettgeri*

The prediction is that, compared to a deletion of drosomycin, a
deletion of Diptericin A should provoke a sharp increase in mortality
upon infection and an increase in PLUD.

```
d_surv_dptA <- read.table(file="Drs_Sk1_SURVIVAL.csv",header=TRUE,sep=";",stringsAsFactors = TRUE)
d_cfu_dptA <- read.table(file="Drs_Sk1_CFU.csv",header=TRUE,sep=";")
d_surv_dptA$Line <- relevel(d_surv_dptA$Line,"Drs")
genotype <- levels(d_surv_dptA$Line)
with(d_surv_dptA,table(Line,Box,Bacteria))
```

```
## , , Bacteria = Pr-1
## 
##      Box
## Line    1   2   5   8   9  11
##   Drs   0   0   0   0   0  50
##   Dpt   0   0 109   0   0   0
## 
## , , Bacteria = Pr-2
## 
##      Box
## Line    1   2   5   8   9  11
##   Drs   0   0   0  53   0   0
##   Dpt   0   0   0   0 114   0
## 
## , , Bacteria = Pr-3
## 
##      Box
## Line    1   2   5   8   9  11
##   Drs 221   0   0   0   0   0
##   Dpt   0 202   0   0   0   0
```

## 3.1 Survival analysis

```
library(survival)
msurv <- survfit(Surv(t,Dead)~Line,data=d_surv_dptA)
dsurv <- data.frame(Line=rep(genotype,msurv$strata),time=msurv$time,proportion=msurv$surv)
dsurv <- rbind(data.frame(Line=genotype,time=0,proportion=1),dsurv)
plot(proportion~time,data=dsurv,type="n",las=1,xlab="Hours post injection",ylab="Proportion surviving",xlim=c(0,48))
color <- c("steelblue","indianred")
names(color) <- genotype
foo<-sapply(split(dsurv,dsurv$Line),function(xx) lines(proportion~time,
                                                  data=xx,type="b",
                                                  cex=0.75,
                                                  pch=21,col=color[as.character(xx$Line)],
                                                  bg=adjustcolor(color[as.character(xx$Line)],alpha.f=0.75)))
points(0,1,pch=21,col="gray",bg="gray",cex=1.25)
n <- with(d_surv_dptA,tapply(Dead,Line,length))
legend("topright",legend=paste(genotype," (N=",n[genotype],")",sep=""),pch=21,col=color,pt.bg=adjustcolor(color,alpha.f=0.75),bty="n")
```

```
require(coxme)
m_full <- coxme(Surv(t,Dead)~Line+(1|Bacteria)+(1|Box),data=d_surv_dptA)
anova(m_full)
```

```
## Analysis of Deviance Table
##  Cox model: response is Surv(t, Dead)
## Terms added sequentially (first to last)
## 
##       loglik  Chisq Df Pr(>|Chi|)    
## NULL -4196.7                         
## Line -4159.7 73.948  1  < 2.2e-16 ***
## ---
## Signif. codes:  0 '***' 0.001 '**' 0.01 '*' 0.05 '.' 0.1 ' ' 1
```

**Conclusion:** suppressing Dipt accelerates mortality
upon infection compared to suppressing Drosomycin.

## 3.2 Analysis of PLUD

Estimation of the PLUD

```
require(CFUfit)
plud_dptA <-  with(d_cfu_dptA,
              loadEstimate(Bacteria,
                      1/(4*Dilution),
                      Volume,
                      sample_ID,
                      maxCFU = 50,
                      ignoreAboveMaxCFU = FALSE,
                      logbase = 10))

pos <- match(plud_dptA$sampleID,d_surv_dptA$sampleID)
plud_dptA$Line        <- d_surv_dptA[pos,"Line"]
plud_dptA$Box         <- d_surv_dptA[pos,"Box"]
plud_dptA$Experiment  <- d_surv_dptA[pos,"Experiment"]
plud_dptA$Bacteria    <- d_surv_dptA[pos,"Bacteria"]
plud_dptA$t           <- d_surv_dptA[pos,"t"]
plud_dptA             <- subset(plud_dptA,!is.na(plud_dptA$Line))
```

Detecting PLUD outliers

```
library(EnvStats)
plud_dptA$outlier <- FALSE
plud_dptA$grpe <- with(plud_dptA,Line)
for(g in levels(plud_dptA$grpe)) {
    l <- plud_dptA$grpe == g & !is.na(plud_dptA$logload)
    test <- try(rosnerTest(plud_dptA$logload[l],k = floor(sum(l,na.rm=TRUE)/2)))
    if("try-error" %in% class(test)) {
        plud_dptA$outlier[l] <- NA
    } else {
        plud_dptA$outlier[which(l)[test$all.stats$Obs.Num[test$all.stats$Outlier]]] <- TRUE
    }
}
sum(plud_dptA$outlier)
```

```
## [1] 5
```

No outliers have been detected.

```
require(beanplot)
require(beeswarm)

gr <- beanplot(logload~Line,
               ylim=c(3.75,7),
               col=as.list(adjustcolor(color,alpha.f=0.75)),
               what=c(F,T,T,F),
               axes=TRUE,
               data=subset(plud_dptA,!outlier))
title(ylab="log_10 PLUD",xlab="D. melanogaster line")
box()
pos <- 1:4; names(pos) <- gr$names
with(plud_dptA,
     beeswarm(logload~Line,
              pwpch = ifelse(outlier,4,21),
              cex=0.75,
              bg="white",add=T))
n <- with(subset(plud_dptA,!is.na(logload)),tapply(logload,factor(Line,levels=genotype),length))
mtext(text=paste0("(N=",as.numeric(n),")"),side=3,line=0,at=1:4)
```

```
require(spaMM)
require(DHARMa)

## first analysis: outliers are not removed
m_full <- fitme(logload~Line+(1|Box)+(1|Bacteria),
                prior.weights=1/(se.logload^2),
                data=plud_dptA)

## Goodness of fit
sim <- simulateResiduals(fittedModel = m_full,refit = TRUE) 
plot(sim) ## significant deviation from normality
```

```
m_full <- fitme(logload~Line+(1|Box)+(1|Bacteria),
                prior.weights=1/(se.logload^2),
                data=subset(plud_dptA,!outlier))

## Goodness of fit
sim <- simulateResiduals(fittedModel = m_full,refit = TRUE) 
plot(sim) ## deviation from normality is marginally significant...
```

```
m_noLine <- fitme(logload~1+(1|Box)+(1|Bacteria),
                prior.weights=1/(se.logload^2),
                data=subset(plud_dptA,!outlier))

#test of Line differences
anova(m_full,m_noLine)
```

```
##      chi2_LR df     p_value
## p_v 10.11344  1 0.001471918
```

**Conclusion** deleting Dipt yields a higher PLUD than
deleting Drosomycin, as the model predicts. The slight deviation from
normality in the last analysis might be a problem, but it is confirmed
by the bootstrap CI below.

## 3.3 PLUD as a function of HR

Computing confidence interval relative to Drs.

```
library(boot)
dtmp <- subset(d_surv_dptA,Line %in% genotype)
dtmp <- dtmp[c("t","Dead","Line","Bacteria","Box")]
dtmp <- na.omit(dtmp)
m <- coxme(Surv(t,Dead)~Line+(1|Box)+(1|Bacteria),data=dtmp)
cox.fun <- function(d) {mtmp <- update(m,data=d); return(mtmp$coef)}
bcox <- censboot(dtmp, cox.fun, R = 499,strata=dtmp$Line) ## sample size per Line must stay constant

ci <- list()
for(l in c("Dpt"))
  ci[[l]] <- boot::boot.ci(bcox,conf=0.95,type="perc",
                           index=match(paste0("Line",l),names(bcox$t0)))

d.lHR <- data.frame(lHR=sapply(ci,function(xx) xx$t0),
                    lHR.inf=sapply(ci,function(xx) xx$percent[4]),
                    lHR.sup=sapply(ci,function(xx) xx$percent[5]))
row.names(d.lHR) <- names(ci)

dtmp <- subset(plud_dptA,!is.na(logload))
m_full <- fitme(logload~Line+(1|Box)+(1|Bacteria),
                prior.weights=1/(se.logload^2),
                data=dtmp)
ci <- list()
for(l in c("Dpt")) ci[[l]] <- confint(m_full,parm=paste0("Line",l),
                                                    boot_args=list(nsim=499, seed=123))
```

```
## Bootstrap replicates:  bootstrap took 42.39 s.
## BOOTSTRAP CONFIDENCE INTERVAL CALCULATIONS
## Based on 499 bootstrap replicates
## 
## CALL : 
## boot.ci(boot.out = list(499, "parametric"), conf = 0.95, type = c("basic", 
## "perc", "norm"), t0 = 0.190292618307475, `str(long numeric 't')` = " num [1:499] 0.186 0.189 0.172 0.146 0.166 ...")
## 
## Intervals : 
## Level      Normal              Basic              Percentile     
## 95%   ( 0.1447,  0.2488 )   ( 0.1436,  0.2492 )   ( 0.1314,  0.2370 )  
## Calculations and Intervals on Original Scale
```

```
d.lPR <- data.frame(lPR=sapply(ci,function(cc) cc$t0),
                    lPR.inf=sapply(ci,function(cc) cc$percent[4]),
                    lPR.sup=sapply(ci,function(cc) cc$percent[5]))
rownames(d.lPR) <- names(ci)
d.lHR.lPR <- cbind(d.lHR,d.lPR)
d.lHR.lPR
```

```
##          lHR   lHR.inf   lHR.sup       lPR  lPR.inf   lPR.sup
## Dpt 0.376774 0.1732743 0.5905618 0.1902926 0.131427 0.2369928
```

Estimated log PLUD ratio as a function of estimated log HR.

```
plot(lPR~lHR,
     data=d.lHR.lPR,
     xlim=c(0,0.75),ylim=c(-0.1,0.3),type="p",las=1,
     xlab="log HR relative to Dpt on",
     ylab="log PLUD ratio relative to Dpt on")
abline(h=0,lty=2)
abline(v=0,lty=2)
with(d.lHR.lPR,segments(lHR.inf,lPR,lHR.sup,lPR,col="indianred"))
with(d.lHR.lPR,segments(lHR,lPR.inf,lHR,lPR.sup,col="indianred"))
points(lPR~lHR,data=d.lHR.lPR,pch=21,col="white",bg="white",cex=2)
points(lPR~lHR,data=d.lHR.lPR,pch=21,
       col="indianred",bg=adjustcolor("indianred",alpha.f=0.75))
points(0,0,pch=21,col="white",bg="white",cex=2)
points(0,0,pch=21,bg=adjustcolor("steelblue",alpha.f=0.75),col="steelblue")
with(d.lHR.lPR,text(lHR,lPR,rownames(d.lHR.lPR),adj=c(-0.5,-1),offset = 1))
text(0,0,"Drs",adj=c(-0.2,-1))
```

# 4 Silencing Diptericin A also increases both susceptibility and the PLUD

To confirm the fact that suppressing Diptericin A increases the PLUD,
we now test the effect of silencing Diptericin A (DiptA), the most
active effector against P. rettgeri. We used for that a RNAi targeting
DiptA (line 53923) which we compared to an inactive RNAi (line 36304).
Compared to the previous approach which uses mutants, the use of RNAi
has the advantage that we can use as a control a lineage which has the
same genetic background than the one in which DpitA is silenced. The
potential drawback is that the RNAi may not completely suppress the
expression of DiptA.

The analysis follows the same global logic as in the previous
sections.

```
d_surv_rnai <- read.table(file="RNAi_dptA_SURVIVAL.csv",header=TRUE,sep=";")
d_surv_rnai$Line <- factor(ifelse(d_surv_rnai$Line==36304,"DptA on","DptA off"),levels=c("DptA on","DptA off"))
d_surv_rnai$sampleID <- factor(with(d_surv_rnai,ifelse(is.na(Plate),NA,as.character(sampleID))))
genotype <- levels(d_surv_rnai$Line)
d_cfu_rnai <- read.table(file="RNAi_dptA_CFU.csv",header=TRUE,sep=";")
```

## 4.1 Survival analysis

```
library(survival)
msurv <- survfit(Surv(t,Dead)~Line,data=d_surv_rnai)
dsurv <- data.frame(Line=rep(genotype,msurv$strata),time=msurv$time,proportion=msurv$surv)
dsurv <- rbind(data.frame(Line=genotype,time=0,proportion=1),dsurv)
plot(proportion~time,data=dsurv,type="n",las=1,xlab="Hours post injection",ylab="Proportion surviving")
color <- c("steelblue","indianred")
names(color) <- genotype
foo<-sapply(split(dsurv,dsurv$Line),function(xx) lines(proportion~time,
                                                  data=xx,type="b",
                                                  cex=0.75,
                                                  pch=21,col=color[as.character(xx$Line)],
                                                  bg=adjustcolor(color[as.character(xx$Line)],alpha.f=0.75)))
points(0,1,pch=21,col="gray",bg="gray",cex=1.25)
n <- with(d_surv_rnai,tapply(Dead,Line,length))
legend("topright",legend=paste(genotype," (N=",n[genotype],")",sep=""),pch=21,col=color,pt.bg=adjustcolor(color,alpha.f=0.75),bty="n")
```

```
require(coxme)
m_full <- coxme(Surv(t,Dead)~Line+(1|Experiment/Box),data=d_surv_rnai)
anova(m_full)
```

```
## Analysis of Deviance Table
##  Cox model: response is Surv(t, Dead)
## Terms added sequentially (first to last)
## 
##       loglik  Chisq Df Pr(>|Chi|)    
## NULL -3136.0                         
## Line -3026.7 218.64  1  < 2.2e-16 ***
## ---
## Signif. codes:  0 '***' 0.001 '**' 0.01 '*' 0.05 '.' 0.1 ' ' 1
```

**Conclusion:** silencing DiptA accelerates massively
mortality upon infection.

## 4.2 Analysis of PLUD

Estimation of the PLUD

```
require(CFUfit)
plud_rnai <-  with(d_cfu_rnai,
              loadEstimate(Bacteria,
                      1/(4*Dilution),
                      Volume,
                      sample_ID,
                      maxCFU = 50,
                      ignoreAboveMaxCFU = FALSE,
                      logbase = 10))

pos <- match(plud_rnai$sampleID,d_surv_rnai$sampleID)
plud_rnai$Line      <- d_surv_rnai[pos,"Line"]
plud_rnai$Box     <- d_surv_rnai[pos,"Box"]
plud_rnai$Experiment     <- d_surv_rnai[pos,"Experiment"]
plud_rnai$t         <- d_surv_rnai[pos,"t"]
plud_rnai <- subset(plud_rnai,!is.na(plud_rnai$Line))
```

Detecting PLUD outliers

```
library(EnvStats)
plud_rnai$outlier <- FALSE
plud_rnai$grpe <- with(plud_rnai,factor(paste(Line,Experiment,Box,sep="_")))
for(g in levels(plud$grpe)) {
    l <- plud_rnai$grpe == g & !is.na(plud_rnai$logload)
    test <- try(rosnerTest(plud_rnai$logload[l],k = floor(sum(l,na.rm=TRUE)/2)))
    if("try-error" %in% class(test)) {
        plud_rnai$outlier[l] <- NA
    } else {
        plud_rnai$outlier[which(l)[test$all.stats$Obs.Num[test$all.stats$Outlier]]] <- TRUE
    }
}
```

```
## Error in rosnerTest(plud_rnai$logload[l], k = floor(sum(l, na.rm = TRUE)/2)) : 
##   There must be at least 3 non-missing finite observations in 'x'
## Error in rosnerTest(plud_rnai$logload[l], k = floor(sum(l, na.rm = TRUE)/2)) : 
##   There must be at least 3 non-missing finite observations in 'x'
## Error in rosnerTest(plud_rnai$logload[l], k = floor(sum(l, na.rm = TRUE)/2)) : 
##   There must be at least 3 non-missing finite observations in 'x'
## Error in rosnerTest(plud_rnai$logload[l], k = floor(sum(l, na.rm = TRUE)/2)) : 
##   There must be at least 3 non-missing finite observations in 'x'
## Error in rosnerTest(plud_rnai$logload[l], k = floor(sum(l, na.rm = TRUE)/2)) : 
##   There must be at least 3 non-missing finite observations in 'x'
## Error in rosnerTest(plud_rnai$logload[l], k = floor(sum(l, na.rm = TRUE)/2)) : 
##   There must be at least 3 non-missing finite observations in 'x'
## Error in rosnerTest(plud_rnai$logload[l], k = floor(sum(l, na.rm = TRUE)/2)) : 
##   There must be at least 3 non-missing finite observations in 'x'
## Error in rosnerTest(plud_rnai$logload[l], k = floor(sum(l, na.rm = TRUE)/2)) : 
##   There must be at least 3 non-missing finite observations in 'x'
```

```
sum(plud_rnai$outlier)
```

```
## [1] 0
```

No outliers have been detected.

```
require(beanplot)
require(beeswarm)

gr <- beanplot(logload~Line,
               ylim=c(4.25,7),
               col=as.list(adjustcolor(color,alpha.f=0.75)),
               what=c(F,T,T,F),
               axes=TRUE,
               data=subset(plud_rnai,!outlier))
title(ylab="log_10 PLUD",xlab="D. melanogaster line")
box()
pos <- 1:4; names(pos) <- gr$names
with(plud_rnai,
     beeswarm(logload~Line,
              pwpch = ifelse(outlier,4,21),
              cex=0.75,
              bg="white",add=T))
n <- with(subset(plud_rnai,!is.na(logload)),tapply(logload,factor(Line,levels=genotype),length))
mtext(text=paste0("(N=",as.numeric(n),")"),side=3,line=0,at=1:4)
```

```
require(spaMM)
require(DHARMa)

## first analysis: outliers are not removed
m_full <- fitme(logload~Line+(1|Experiment/Box),
                prior.weights=1/(se.logload^2),
                data=plud_rnai)

## Goodness of fit
sim <- simulateResiduals(fittedModel = m_full,refit = TRUE) 
plot(sim) ## all is good!
```

```
m_noLine <- fitme(logload~1+(1|Experiment/Box),
                prior.weights=1/(se.logload^2),
                data=subset(plud_rnai,!outlier))

#test of Line differences
anova(m_full,m_noLine)
```

```
##      chi2_LR df     p_value
## p_v 8.421592  1 0.003707909
```

**Conclusion:** silencing DiptA does increase the PLUD,
as the model predicts.

## 4.3 PLUD as a function of HR

Computing confindence interval relative to BOM.

```
library(boot)
dtmp <- subset(d_surv_rnai,Line %in% genotype)
dtmp <- dtmp[c("t","Dead","Line","Experiment","Box")]
dtmp <- na.omit(dtmp)
m <- coxme(Surv(t,Dead)~Line+(1|Experiment/Box),data=dtmp)
cox.fun <- function(d) {mtmp <- update(m,data=d); return(mtmp$coef)}
bcox <- censboot(dtmp, cox.fun, R = 499,strata=dtmp$Line) ## sample size per Line must stay constant

ci <- list()
for(l in c("DptA off"))
  ci[[l]] <- boot::boot.ci(bcox,conf=0.95,type="perc",
                           index=match(paste0("Line",l),names(bcox$t0)))

d.lHR <- data.frame(lHR=sapply(ci,function(xx) xx$t0),
                    lHR.inf=sapply(ci,function(xx) xx$percent[4]),
                    lHR.sup=sapply(ci,function(xx) xx$percent[5]))
row.names(d.lHR) <- names(ci)

dtmp <- subset(plud_rnai,!is.na(logload))
m_full <- fitme(logload~Line+(1|Experiment/Box),
                prior.weights=1/(se.logload^2),
                data=dtmp)
ci <- list()
for(l in c("DptA off")) ci[[l]] <- confint(m_full,parm=paste0("Line",l),
                                                  boot_args=list(nsim=499, seed=123))
```

```
## Bootstrap replicates:  bootstrap took 33.46 s.
## BOOTSTRAP CONFIDENCE INTERVAL CALCULATIONS
## Based on 499 bootstrap replicates
## 
## CALL : 
## boot.ci(boot.out = list(499, "parametric"), conf = 0.95, type = c("basic", 
## "perc", "norm"), t0 = 0.201712282567498, `str(long numeric 't')` = " num [1:499] 0.2 0.195 0.162 0.238 0.213 ...")
## 
## Intervals : 
## Level      Normal              Basic              Percentile     
## 95%   ( 0.1329,  0.2747 )   ( 0.1329,  0.2772 )   ( 0.1262,  0.2705 )  
## Calculations and Intervals on Original Scale
```

```
d.lPR <- data.frame(lPR=sapply(ci,function(cc) cc$t0),
                    lPR.inf=sapply(ci,function(cc) cc$percent[4]),
                    lPR.sup=sapply(ci,function(cc) cc$percent[5]))
rownames(d.lPR) <- names(ci)
d.lHR.lPR <- cbind(d.lHR,d.lPR)
d.lHR.lPR
```

```
##               lHR  lHR.inf  lHR.sup       lPR  lPR.inf   lPR.sup
## DptA off 1.462996 1.222169 1.703617 0.2017123 0.126206 0.2705496
```

Estimated log PLUD ratio as a function of estimated log HR.

```
plot(lPR~lHR,
     data=d.lHR.lPR,
     xlim=c(0,2.5),ylim=c(-0.1,0.3),type="p",las=1,
     xlab="log HR relative to DptA on",
     ylab="log PLUD ratio relative to DptA on")
abline(h=0,lty=2)
abline(v=0,lty=2)
with(d.lHR.lPR,segments(lHR.inf,lPR,lHR.sup,lPR,col="indianred"))
with(d.lHR.lPR,segments(lHR,lPR.inf,lHR,lPR.sup,col="indianred"))
points(lPR~lHR,data=d.lHR.lPR,pch=21,col="white",bg="white",cex=2)
points(lPR~lHR,data=d.lHR.lPR,pch=21,
       col="indianred",bg=adjustcolor("indianred",alpha.f=0.75))
points(0,0,pch=21,col="white",bg="white",cex=2)
points(0,0,pch=21,bg=adjustcolor("steelblue",alpha.f=0.75),col="steelblue")
with(d.lHR.lPR,text(lHR,lPR,rownames(d.lHR.lPR),adj=c(-0.5,-1),offset = 1))
text(0,0,"DptA on",adj=c(-0.2,-1))
```

# 5 Silencing Catalase increases susceptibility but decreases the PLUD

We tested our prediction that reduced tolerance should increase
susceptibility and increase the PLUD by testing the effect of silencing
Catalase. Catalase is among several enzymes involved in scavenging
oxygen free radicals and protecting cells from reactive oxygen species
(ROS). The activity of Catalases into reducing ROS has been shown in
Drosophila infections (Ha 2005) and mosquito systemic infections (DeJong
2007), making it a strong candidate for a tolerance gene. As in the
previous experiment we have RNAi lines in this experiment.

The analysis follows the same global logic as in the previous
sections.

```
d_surv_catalase <- read.table(file="RNAi_catalase_SURVIVAL.csv",header=TRUE)
d_plud_catalase <- read.table(file="RNAi_catalase_PLUD.csv",header=TRUE)
d_surv_catalase$Genotype <- factor(d_surv_catalase$Genotype,c("attP2","UAS-Cat-iR"))
d_plud_catalase$Genotype <- factor(d_plud_catalase$Genotype,c("attP2","UAS-Cat-iR"))
genotype <- levels(d_surv_catalase$Genotype)
```

## 5.1 Survival analysis

```
library(survival)
msurv <- survfit(Surv(Time_to_death_hours,Censor)~Genotype,data=d_surv_catalase)
dsurv <- data.frame(Line=rep(genotype,msurv$strata),time=msurv$time,proportion=msurv$surv)
dsurv <- rbind(data.frame(Line=genotype,time=0,proportion=1),dsurv)
plot(proportion~time,data=dsurv,type="n",las=1,xlab="Hours post injection",ylab="Proportion surviving")
color <- c("steelblue","indianred")
names(color) <- genotype
foo<-sapply(split(dsurv,dsurv$Line),function(xx) lines(proportion~time,
                                                  data=xx,type="b",
                                                  cex=0.75,
                                                  pch=21,col=color[as.character(xx$Line)],
                                                  bg=adjustcolor(color[as.character(xx$Line)],alpha.f=0.75)))
points(0,1,pch=21,col="gray",bg="gray",cex=1.25)
n <- with(d_surv_catalase,tapply(Censor,Genotype,length))
legend("topright",legend=paste(genotype," (N=",n[genotype],")",sep=""),pch=21,col=color,pt.bg=adjustcolor(color,alpha.f=0.75),bty="n")
```

```
require(coxme)
m_full <- coxme(Surv(Time_to_death_hours,Censor)~Genotype +(1|Day_of_injection),
             data=d_surv_catalase)
anova(m_full)
```

```
## Analysis of Deviance Table
##  Cox model: response is Surv(Time_to_death_hours, Censor)
## Terms added sequentially (first to last)
## 
##           loglik  Chisq Df Pr(>|Chi|)    
## NULL     -1513.4                         
## Genotype -1430.6 165.54  1  < 2.2e-16 ***
## ---
## Signif. codes:  0 '***' 0.001 '**' 0.01 '*' 0.05 '.' 0.1 ' ' 1
```

**Conclusion:** silencing Catalase accelerates massively
mortality upon infection.

## 5.2 Analysis of PLUD

Experiments have ran using a spiroplater. Cell concentration is
therefore estimated through the specific method of this device.
Detecting PLUD outliers

```
library(EnvStats)
d_plud_catalase$outlier <- FALSE
d_plud_catalase$grpe <- with(d_plud_catalase,paste(Genotype,Day_of_injection,sep="_"))
for(g in levels(d_plud_catalase$grpe)) {
    l <- d_plud_catalase$grpe == g & !is.na(d_plud_catalase$log_plud)
    test <- try(rosnerTest(d_plud_catalase$log_plud[l],k = floor(sum(l,na.rm=TRUE)/2)))
    if("try-error" %in% class(test)) {
        d_plud_catalase$outlier[l] <- NA
    } else {
        d_plud_catalase$outlier[which(l)[test$all.stats$Obs.Num[test$all.stats$Outlier]]] <- TRUE
    }
}
with(d_plud_catalase,table(Genotype,outlier))
```

```
##             outlier
## Genotype     FALSE
##   attP2         38
##   UAS-Cat-iR    39
```

No outliers have been detected.

```
require(beanplot)
require(beeswarm)

gr <- beanplot(log_plud~Genotype,
               ylim=c(6.5,8.5),
               col=as.list(adjustcolor(color,alpha.f=0.75)),
               what=c(F,T,T,F),
               axes=TRUE,
               data=d_plud_catalase)
title(ylab="log_10 PLUD",xlab="D. melanogaster line")
box()
pos <- 1:4; names(pos) <- gr$names
with(d_plud_catalase,
     beeswarm(log_plud~Genotype,
              pwpch = ifelse(outlier,4,21),
              cex=0.75,
              bg="white",add=T))
n <- with(subset(d_plud_catalase,!is.na(log_plud)),tapply(log_plud,factor(Genotype,levels=genotype),length))
mtext(text=paste0("(N=",as.numeric(n),")"),side=3,line=0,at=1:4)
```

```
require(spaMM)
require(DHARMa)

## first analysis: outliers are not removed
m_full <- fitme(log_plud~Genotype+(1|Day_of_injection),
                resid.model = ~Genotype,
                data=d_plud_catalase)

## Goodness of fit
sim <- simulateResiduals(fittedModel = m_full,refit = TRUE) 
plot(sim) ## all is good!
```

```
m_noLine <- fitme(log_plud~1+(1|Day_of_injection),
                resid.model = ~Genotype,
                data=d_plud_catalase)

#test of Line differences
anova(m_full,m_noLine)
```

```
##     chi2_LR df      p_value
## p_v  23.326  1 1.367393e-06
```

**Conclusion:** silencing Catalase does decrease the
PLUD. This suggest that Catalase is part of what permit *D.
melanogaster* to tolerate bacterial diseases.

## 5.3 PLUD as a function of HR

Computing confindence interval relative to mock RNAi.

```
library(boot)
dtmp <- d_surv_catalase[,c("Time_to_death_hours","Censor","Genotype","Day_of_injection")]
dtmp <- na.omit(dtmp)
m <- coxme(Surv(Time_to_death_hours,Censor)~Genotype+(1|Day_of_injection),data=dtmp)
cox.fun <- function(d) {mtmp <- update(m,data=d); return(mtmp$coef)}
bcox <- boot::censboot(dtmp, cox.fun, R = 499,strata=dtmp$Genotype) ## sample size per Line must stay constant

ci <- list()
for(l in c("UAS-Cat-iR"))
  ci[[l]] <- boot::boot.ci(bcox,conf=0.95,type="perc",
                           index=match(paste0("Genotype",l),names(bcox$t0)))

d.lHR <- data.frame(lHR=sapply(ci,function(xx) xx$t0),
                    lHR.inf=sapply(ci,function(xx) xx$percent[4]),
                    lHR.sup=sapply(ci,function(xx) xx$percent[5]))
row.names(d.lHR) <- names(ci)

dtmp <- subset(d_plud_catalase,!is.na(log_plud))
m_full <- fitme(log_plud~Genotype+(1|Day_of_injection),
                resid.model = ~Genotype,
                data=dtmp)
ci <- list()
for(l in c("UAS-Cat-iR")) ci[[l]] <- confint(m_full,parm=paste0("Genotype",l),
                                                  boot_args=list(nsim=499, seed=123))
```

```
## Bootstrap replicates:  bootstrap took 30.23 s.
## BOOTSTRAP CONFIDENCE INTERVAL CALCULATIONS
## Based on 499 bootstrap replicates
## 
## CALL : 
## boot.ci(boot.out = list(499, "parametric"), conf = 0.95, type = c("basic", 
## "perc", "norm"), t0 = -0.176893786925361, `str(long numeric 't')` = " num [1:499] -0.199 -0.165 -0.164 -0.214 -0.162 ...")
## 
## Intervals : 
## Level      Normal              Basic              Percentile     
## 95%   (-0.2392, -0.1164 )   (-0.2422, -0.1181 )   (-0.2357, -0.1116 )  
## Calculations and Intervals on Original Scale
```

```
d.lPR <- data.frame(lPR=sapply(ci,function(cc) cc$t0),
                    lPR.inf=sapply(ci,function(cc) cc$percent[4]),
                    lPR.sup=sapply(ci,function(cc) cc$percent[5]))
rownames(d.lPR) <- names(ci)

d.lHR.lPR <- cbind(d.lHR,d.lPR)
d.lHR.lPR
```

```
##                 lHR  lHR.inf  lHR.sup        lPR    lPR.inf   lPR.sup
## UAS-Cat-iR 1.810129 1.521728 2.246199 -0.1768938 -0.2356705 -0.111559
```

Estimated log PLUD ratio as a function of estimated log HR.

```
plot(lPR~lHR,
     data=d.lHR.lPR,
     xlim=c(0,2.5),ylim=c(-0.3,0.1),type="p",las=1,
     xlab="log HR relative to Catalase on",
     ylab="log PLUD ratio relative to Calase on")
abline(h=0,lty=2)
abline(v=0,lty=2)
with(d.lHR.lPR,segments(lHR.inf,lPR,lHR.sup,lPR,col="indianred"))
with(d.lHR.lPR,segments(lHR,lPR.inf,lHR,lPR.sup,col="indianred"))
points(lPR~lHR,data=d.lHR.lPR,pch=21,col="white",bg="white",cex=2)
points(lPR~lHR,data=d.lHR.lPR,pch=21,
       col="indianred",bg=adjustcolor("indianred",alpha.f=0.75))
points(0,0,pch=21,col="white",bg="white",cex=2)
points(0,0,pch=21,bg=adjustcolor("steelblue",alpha.f=0.75),col="steelblue")
with(d.lHR.lPR,text(lHR,lPR,rownames(d.lHR.lPR),adj=c(-0.5,-1),offset = 1))
text(0,0,"Catalase on",adj=c(-0.2,-1))
```
